# Role of osteopontin in tumour progression

**DOI:** 10.1038/sj.bjc.6601839

**Published:** 2004-04-27

**Authors:** S R Rittling, A F Chambers

**Affiliations:** 1 Department of Genetics, Rutgers University, Piscataway, NJ, USA; 2 Departments of Oncology and Pathology, University of Western Ontario, London, Ontario, Canada

**Keywords:** tumorigenesis, metastasis, prognosis, tumour marker

## Abstract

Since its first identification as a transformation-associated protein, osteopontin (OPN) has been recognised as important in the processes of tumorigenicity and metastasis. Here, we review the evidence that OPN might be considered as a candidate prognostic marker in human cancer. In animal systems, evidence from cell injection experiments and genetically manipulated mice suggest an important but complex role for the protein in tumour progression. Moreover, studies in a variety of human cancers associate high levels of OPN expression in tumours or in blood with more advanced cancers. The mechanism of action of OPN in promoting cancer is still unclear, and we consider aspects of OPN biology that can complicate interpretation of human studies. Nevertheless, growing evidence supports a role for OPN as a potential prognostic factor for various human cancers.

The first description of the protein that would eventually come to be known as osteopontin (OPN) was as a marker of transformation of epithelial cells (Senger *et al*, 1979). Thus it is appropriate that 25 years on, there is considerable interest in the role of OPN in human tumorigenesis, both as a marker of malignancy as well as a candidate for testing as a prognostic factor. In this review, we consider the current evidence for both these roles as well as evidence from both animal models and *in vitro* experiments supporting the idea that OPN acts to facilitate tumour development.

## OPN FUNCTION

In the two and a half decades since its initial description, OPN protein or mRNA has been identified in a series of independent biological models. Most notably, its identification as a key noncollagenous bone matrix protein earned it the name osteopontin, but the protein has also been shown to have an important role in diverse systems ranging from the immune system, where OPN regulates cytokine production and cell trafficking, to the vascular system, where it inhibits ectopic mineralisation and macrophage accumulation (Rittling *et al*, 2003). The protein is secreted, variably phosphorylated and accumulates in bodily fluids; its strong affinity for hydroxyapatite leads to its accumulation in bone and other sites of mineralisation. While much of the molecule appears to lack secondary structure, a central region contains sequences that bind to as many as seven different integrins, including *α*_v_*β*_3_ and *β*_5_, and a series of *β*_1_-containing integrins: the protein has a cryptic *α*_9_*β*_1_ site that is functional only after protease cleavage, implying a unique role for OPN fragments (Yamamoto *et al*, 2003). Osteopontin interacts with cells through these integrins as well as through CD44, while an intracellular role for OPN has also been suggested wherein OPN binds CD44 on the inside face of the cell membrane (Sodek *et al*, 2000).

*In vitro* studies have shown that OPN has several important functions in cells. Early on, it was recognised that OPN had adhesive activity, confirmed by the observation that its receptors all mediate cell adhesion. An additional well-characterised function of OPN is in regulating migration: not only is it chemotactic for many cell types but also OPN-deficient cells are hypomotile (Zhu *et al*, 2003), suggesting that the protein plays an intrinsic role in migration. The protein regulates cytokine production by macrophages, and in several diverse systems, it has been shown to act as a survival factor (Denhardt *et al*, 2001). The precise molecular mechanism of OPN's action in different disease states, however, remains poorly defined.

### OPN in tumorigenesis: animal studies

It is clear from numerous studies in cultured cells over the last 10 years that OPN expression renders cells more tumorigenic and/or metastatic. In antisense experiments, downregulation of OPN expression reduced growth in soft agar, growth of injected cells as primary tumours or experimental metastasis (eg Behrend *et al*, 1994; see also, Denhardt *et al*, 2001 for review). Recently, downregulation of OPN was shown to reduce tumorigenicity of HGF-transformed cells, implicating OPN in the mechanism of transformation by HGF (Ariztia *et al*, 2003). In experiments examining parallel ras-transformed 3T3 cell lines from wild-type (WT) and OPN-deficient mice, the transformed properties of the OPN-deficient cells were uniformly attenuated as compared to WT (Wu *et al*, 2000): both growth of primary tumours arising from cells injected subcutaneously as well as experimental metastasis were reduced in OPN-deficient cells.

Compelling evidence on the role of OPN specifically in metastasis came from experiments in which transfection of tumorigenic, but not metastatic, rat mammary epithelial cells with DNA fragments that induced OPN expression converted the cells from benign tumorigenic cells to fully metastatic (review: Barraclough *et al*, 1998). Interestingly, the DNA fragments responsible for these effects did not code directly for OPN protein, or even for any protein, rather they acted as competitors for binding of the transcription factor TCF-4, which suppresses OPN expression. Finally, while studies correlating elevated OPN expression with increased malignancy of a variety of cell lines are too numerous to list here, recently several groups have noted elevated OPN expression associated with the metastatic phenotype following selection and expression profiling of metastatic and nonmetastatic variants of human breast cancer cells (Urquidi *et al*, 2002; Kang *et al*, 2003).

Experiments evaluating tumorigenesis in OPN-knockout mice directly have yielded disparate results, possibly because OPN expressed by normal tissues and tumours may have differential functional effects. In a squamous carcinoma model, primary skin tumours were larger and more malignant in OPN-deficient mice as compared to their WT counterparts. Spontaneously arising lung metastases were also more numerous in OPN-deficient mice as compared to WT, but the metastases were smaller (Crawford *et al*, 1998). An important role for OPN expressed by host cells was implicated in this model, as host cells in WT mice expressed significant amounts of OPN.

On the other hand, the growth of primary mammary tumours developing spontaneously either in transgenic mice carrying c-*myc* and v-Ha-*ras* oncogenes expressed in the mammary gland or in mice treated with DMBA in the presence of progesterone was unaffected by OPN status: primary tumours developed with similar kinetics in both WT and OPN−/− mice. However, neither of these tumours metastasised at a high enough frequency to allow an evaluation of the effect of OPN on metastasis (Feng and Rittling, 2000; Chen and Rittling, 2003). Finally, in an experimental metastasis model, using melanoma cells that weakly expressed OPN, the number of metastases at a variety of sites was suppressed in the OPN-deficient mice as compared to those in WT (Nemoto *et al*, 2001). Taken together, these results suggest that the role of OPN in tumour development is complex and may be affected by a variety of parameters, including tumour type and experimental system: in turn, these parameters may reflect a role of the tumour microenvironment in determining the effects of OPN. Since OPN can be produced by many cell types in a tumour microenvironment, including tumour cells themselves, activated immune cells, remodelling vasculature, and bone cells, in the case of tumours growing in bone; it may be that OPN from different sources mediate different effects. For instance, OPN originating from different cellular sources may have differential post-translational modifications and/or may be differentially cleaved, suggesting possible differential functions.

### OPN expression in human tumours: potential utility as a tumour marker

Following the identification of OPN as a phosphoprotein secreted by transformed cells in culture (Senger *et al*, 1979), OPN mRNA and protein were shown in histological sections of several types of human cancer to be elevated relative to normal tissue (Brown *et al*, 1994). In this study, OPN RNA was found to be produced primarily by tumour-associated macrophages rather than tumour cells themselves. However, Tuck *et al* (1998), examined a series of tumours from 154 lymph node-negative breast cancer patients. Osteopontin RNA and protein were detected in both tumour cells and infiltrating inflammatory cells: host immune cells were positive for OPN protein in 70% of tumours, while only 26% of tumours were positive for OPN immunostaining in the breast tumour cells themselves. Interestingly, OPN positivity specifically in tumour cells correlated with patient survival, while OPN in host cells was too common to provide prognostic information. This study supported the idea that multiple cell types in a tumour microenvironment may produce OPN, including tumour cells and host infiltrating cells, but that these sources of OPN may be functionally distinct.

OPN has been detected in a growing number of human tumour types, including lung, breast, prostate, gastric, oesophageal, ovarian and glioma, by immunohistochemistry on tumour tissue sections, quantification of OPN RNA from tumour tissue or in expression array studies from tumour tissues (reviews: Furger *et al*, 2001; Tuck and Chambers, 2001). In some of these studies, clinical and patient outcome data are available, enabling OPN expression to be assessed as a potential marker of tumour progression. In a series of 25 lung tumour specimens, OPN protein and RNA were elevated in tumour tissue, relative to normal lung tissue, and OPN immunopositivity was statistically significantly associated with patient survival (Chambers *et al*, 1996). In the study described above by Tuck *et al* (1998), examining tumours from lymph node-negative breast cancer patients, OPN protein detected specifically in the tumour cells in breast tumours correlated significantly with disease free- and overall survival in these patients. Consistent with this finding is a case report of bilateral mammary carcinomas (Tuck *et al*, 1997), in which OPN tumour cell immunopositivity, as well as p53 immunopositivity, were associated with the tumour that recurred locally and progressed to form metastases in the liver and bone. In a recent study of 68 breast tissue samples from primary tumours, nodal metastases, fibroadenomas and normal tissue, OPN protein and RNA were elevated in malignant *vs* benign/normal tissues, although no differences in RNA levels quantified by RT–PCR were found when tumours with *vs* without metastases were compared (Wang-Rodriguez *et al*, 2003). Finally, OPN immunopositivity in a group of 333 breast cancer patients demonstrated a negative correlation with survival (Rudland *et al*, 2002). Given the common finding of OPN in nontumour (infiltrating) cells, reported by Tuck *et al* (1998), it may be that OPN protein detection, when limited to quantification from only the tumour cells, may provide more useful predictive information than RNA levels quantified from tumour tissue, at least for breast cancer.

In contrast, in a study of a series of 240 hepatocellular carcinomas, elevated OPN RNA levels were associated with high grade, late stage and early recurrence, which are all associated with poor patient prognosis (Pan *et al*, 2003). In an expression array study of 60 colorectal tumours, representing a range of stages from adenomas, Astler Collier stages B, C and D, and liver metastases, OPN was identified as the lead marker that was most consistently upregulated with tumour progression (Agrawal *et al*, 2002). Osteopontin RNA and protein in tumour tissue have also been shown to have a potential prognostic value in ovarian cancer, with OPN levels being higher in tumour tissue than in normal or benign tissue (Kim *et al*, 2002). Recently, Coppola *et al* (2004) used tissue arrays to assess OPN protein levels in 350 tumours from 23 body sites compared with 113 normal tissues. In that study, OPN was found to be elevated in tumours, relative to normal tissues. Osteopontin also was found to correlate significantly overall with tumour stage, when considering all tumour sites and to correlate with tumour stage for several sites individually, including bladder, colon, kidney, larynx, mouth and salivary gland (Coppola *et al*, 2004).

OPN RNA and protein thus have been found to be overexpressed in a number of human tumour types, relative to normal tissue. This is perhaps not surprising, since OPN expression can be induced by the *ras* oncogene (Denhardt *et al*, 2003) and the *ras* pathway is activated either directly or indirectly in a high proportion of human tumours (Bos, 1989; Clark and Der, 1995). In some cases, OPN overexpression has been shown to be associated directly with poor patient prognosis or with other indicators of poor prognosis. There also is some suggestion that OPN from different sources may have different prognostic value. As noted above, Tuck *et al* (1998) found that OPN immunopositivity within breast tumour cells was associated with poor patient survival, whereas OPN in infiltrating host cells in the tumours was common and not associated with survival. It should be noted that OPN is not specific to tumours, but is expressed by a number of other tissues, under normal or pathological conditions, and this must be considered in any studies assessing the utility of OPN as a marker of prognosis in cancer. While further studies, using large numbers of samples associated with clinical and patient outcome data, will be needed to determine the prognostic value of OPN in specific human cancers, studies to date support the hypothesis that OPN detected within tumour cells has a potential utility as a prognostic marker.

### OPN expression in human tumours: potential utility as a blood marker

In addition to being present in tumours and some normal tissues, OPN also is found in bodily fluids. By Western blotting, OPN blood levels appeared to be elevated in a small number of patients with various cancers (Senger *et al*, 1988). Bautista *et al* (1996) developed the first ELISA able to quantify OPN levels in blood plasma and using this assay they measured baseline levels in a series of normal women. Singhal *et al* (1997) then used this ELISA to quantify OPN plasma levels in 70 women with metastatic breast cancer compared to healthy women and women on well follow-up for cancer. Osteopontin plasma levels were significantly elevated in women with metastatic breast cancer, relative to either control group (*P*<0.001). Furthermore, elevated OPN levels were significantly associated with patient survival as well as with increased numbers of metastatic sites.

A number of recent studies have confirmed these initial findings and have extended them to other cancer types. Fedarko *et al* (2001) measured OPN serum levels in patients with breast, colon, lung or prostate cancer compared with normal serum, and found elevated OPN levels in all tumour types except colon cancer. No clinical or outcome data on the patients in this study were available. In a prospective study, Hotte *et al* (2002) examined OPN plasma levels in a series of 100 men with hormone refractory prostate cancer. Osteopontin levels were found to correlate negatively and independently with patient survival (*P*=0.029). In these patients there also was a statistically significant correlation between OPN levels and the presence of metastases to bone (*P*=0.024). Plasma OPN also has been examined as a potential prognostic marker in head and neck cancers (Le *et al*, 2003). Osteopontin levels were elevated in these patients when compared with normal control samples: these levels correlated with relapse-free and overall survival in the patients. Finally, Kim *et al* (2002) measured OPN in plasma samples from 144 women being assessed for possible ovarian cancer compared with 107 normal control samples. Plasma OPN levels were elevated in the group of 51 women with ovarian cancer (*P*<0.001) compared with healthy women or women with benign ovarian disease.

Taken together, this growing list of studies suggests that OPN blood levels have a potential as a prognostic or diagnostic marker in prostate, breast, head and neck, and likely other cancers. It should be noted, however, that OPN is unlikely to be a blood marker that is specific to cancer. Osteopontin levels are elevated in other conditions including sepsis, kidney disease and cardiovascular disease, and OPN blood levels in these conditions has not been thoroughly evaluated. Even in patients known to have cancer, OPN blood levels may be elevated due to noncancer causes, which must be considered in evaluating the results. Clearly, larger prospective trials will be needed to assess the ability of plasma OPN to provide prognostic information or indications of treatment responses.

### Mechanism of action of OPN in regulating tumour growth

The mechanisms by which OPN may enhance malignancy are still unclear. However, several mechanisms have been suggested through studies in cultured cells. First, the ability of cells to grow in soft agar or in the absence of adhesion is closely associated with tumorigenicity. Several lines of evidence suggest that OPN enhances growth of transformed cells in suspension, including experiments with inducible OPN (Wu *et al*, 2000) as well as in JB6 cells, in which the addition of OPN to cells increases their ability to grow in soft agar (Chang *et al*. 2003). Secondly, the ability of cells to migrate may be directly tied to their tumorigenicity and OPN clearly participates in pathways regulating migration in diverse cell types including osteoclasts, fibroblasts, macrophages and tumour cells (Tuck *et al*, 2000). Interestingly, in macrophages, OPN regulates migration toward some chemokines but not others, suggesting that the protein may function in a subset of migratory pathways (Zhu *et al*, 2003): perhaps, this observation may help to explain some of the diverse effects of OPN in different tumour systems. Invasiveness is clearly related to migration, but not only do cells need to be motile to invade, they also need to degrade the extracellular matrix. Several studies suggest that OPN increases invasiveness by inducing proteinases, particularly uPA (Tuck *et al*, 1999; Das *et al*, 2003). Finally, recent experiments suggest that OPN acts in concert with several growth factors, including HGF (Medico *et al*, 2001) and EGF (Tuck *et al*, 2003), to induce malignant properties. Again, these observations suggest that OPN may have different effects in different tumours depending on the growth factor milieu.

OPN has also been implicated in the process of angiogenesis, particularly as it is a high-affinity ligand for the *α*_v_*β*_3_ integrin, which is highly expressed on some endothelial cells. Signalling through the *α*_v_*β*_3_ is critical for endothelial cell survival and indeed OPN when immobilised on a surface enhances survival of endothelial cells (Scatena *et al*, 1998). While there have been sporadic reports of OPN functioning to promote angiogenesis, firm evidence linking the protein to vessel development in the *in vivo* systems has been scarce. Recent data demonstrating that OPN accelerates blood vessel formation in matrigel and chorioallantoic membrane assays in the presence of FGF-2, however, suggest that OPN may act in concert with other proangiogenic molecules to enhance angiogenesis (Leali *et al*, 2003). Again, these results underscore the idea that the exact function of OPN in any given situation may be determined by interactions with other factors in the microenvironment.

## CONCLUSIONS

Studies *in vitro* and in animal models of cancer have clearly indicated that OPN can function to regulate tumour growth and progression. Numerous reports of elevated OPN expression in human cancers support the idea that OPN should be considered as a potential prognostic marker for a variety of human cancers. There is clearly a need for larger, well-designed prospective studies to evaluate definitively the utility of OPN as a tumour as well as blood marker of tumour progression. These experiments should take into account that multiple cell types can express OPN and that blood levels of the protein thus may be elevated due to noncancer causes. Finally, studies testing the prognostic importance of coexpression of OPN and other growth factors could yield important new mechanisms of evaluating human cancers.
